# Clinical Factors Associated With Failure of Uterine Compression Sutures in Postpartum Hemorrhage During Cesarean Delivery: A Systematic Review

**DOI:** 10.7759/cureus.117804

**Published:** 2026-10-12

**Authors:** Fatema Yousif Abusin, Abeer Ebaid Mahdi Gabreldaar, Ayodele Adegbite Olaleye, Raim Chaar, Saishte Mahajan, Ayesha Pervaiz, Hanady ME M Osman, Amani Seifeldin Mahmoud Khalifa

**Affiliations:** 1 Obstetrics and Gynaecology, Queens Medical Centre, Nottingham University Hospitals NHS Trust, Nottingham, GBR; 2 Obstetrics and Gynaecology, Women's Health Specialist Hospital, Ministry of National Guard Health Affairs (MNGHA), Riyadh, SAU; 3 Obstetrics and Gynaecology, Hertford County Hospital, East and North Hertfordshire Teaching NHS Trust, Hertford, GBR; 4 Quality Improvement and Patient Safety, Najran Armed Forces Hospital, Ministry of Defense Health Services, Najran, SAU; 5 Obstetrics and Gynaecology, Ahfad University for Women, Khartoum, SDN

**Keywords:** b-lynch, cesarean delivery, placenta accreta spectrum, postpartum hemorrhage, prognostic factors, treatment failure, uterine compression sutures

## Abstract

Uterine compression sutures can preserve the uterus during refractory postpartum hemorrhage, but clinical factors associated with failure are less well established than overall success rates. This review examined evidence specifically applicable to cesarean delivery. PubMed/MEDLINE (Medical Literature Analysis and Retrieval System Online), Embase, Scopus, and Web of Science were searched without a lower publication-date restriction. Eligible studies were peer-reviewed primary investigations of therapeutic uterine compression sutures with cesarean-specific clinical-factor information linked to treatment outcomes. Additional hemostatic treatment and hysterectomy were considered separate endpoints. Risk of bias was assessed using the Quality in Prognosis Studies (QUIPS) tool, and findings were synthesized narratively, supplemented by study-level effect estimates and an exploratory pooled analysis of event proportions. Of 1,010 identified records, 374 remained after deduplication; of these, 76 reports underwent full-text assessment, and finally four studies were included. Their original cohorts contained 105 women, of whom 102 underwent cesarean delivery. Two studies were rated at low overall risk of bias and two at high risk. Placental pathology was the principal directly evaluable clinical factor, although placenta previa and suspected placenta accreta spectrum represented distinct exposures; escalation was more frequent with suspected placenta accreta spectrum. Evidence for other factors was sparse, and factor-specific estimates were not pooled. In an exploratory random-effects analysis, immediate hemorrhage-related hysterectomy occurred in 21.7% (95% CI 14.7-30.8%) of treated cesarean cases. The evidence supports attention to hemorrhage etiology and a distinction between escalation of treatment and loss of the uterus. It does not establish a validated cesarean-specific prediction model or a reliable threshold for abandoning compression sutures.

## Introduction and background

Postpartum hemorrhage (PPH) is an important cause of maternal morbidity and can progress rapidly from persistent bleeding to transfusion, organ dysfunction, and emergency surgery. Population-based studies demonstrate that its burden involves both the frequency of hemorrhage and the increasing need for intensive treatment, emphasizing the importance of timely recognition and an effective sequence of interventions [1,2]. Treatment must address the bleeding source while maintaining circulation and coagulation. The WOMAN (World Maternal Antifibrinolytic) trial established a reduction in death due to bleeding with early tranexamic acid treatment [3], while the E-MOTIVE trial demonstrated the value of an early-detection and treatment bundle after vaginal birth [4]. Neither trial identifies which patients will fail uterine compression sutures during cesarean delivery, but both underline why treatment selection and escalation must be considered within a time-sensitive care pathway.

Uterine compression sutures offer a mechanical approach to controlling bleeding while retaining the uterus. The original B-Lynch technique [5], the Hayman modification [6], and the multiple-square technique described by Cho et al. [7] differ in their placement and relationship to the uterine cavity. These differences matter because a suture applied to the uterine body may not address every source of lower-segment bleeding. Hwu and colleagues described parallel vertical sutures directed at hemorrhage associated with placenta previa or accreta during cesarean delivery [8]. The technical diversity of compression sutures therefore creates both therapeutic options and difficulty in comparing outcomes: the same procedure name may conceal differences in placement, concurrent vessel ligation, and the stage of hemorrhage at which treatment was attempted.

An overall percentage of successful sutures does not identify the clinical factors associated with failure. A patient may need an additional uterus-preserving intervention without undergoing hysterectomy, and delayed hysterectomy for infection or ischemic injury represents a different outcome from uncontrolled intraoperative bleeding. Moreover, predictors of developing PPH cannot automatically be assumed to predict failure after a compression suture has already been placed. The distinction is particularly important when interpreting treatment studies with different delivery routes or heterogeneous surgical sequences [5-8]. The objective of this review was to examine clinical factors associated with failure of therapeutic uterine compression sutures during cesarean delivery, separate directly applicable findings from indirect evidence, and identify the methodological requirements for clinically useful prognostic research.

## Review

Methodology

Reporting Framework

This systematic review was reported using the Preferred Reporting Items for Systematic Reviews and Meta-Analyses (PRISMA) 2020 framework [9].

Eligibility Criteria

The Population, Intervention or Exposure, Comparator, Outcomes, and Study design (PICOS) criteria were used to structure eligibility [10] and are presented in Table 1. Because the question concerns prognosis after treatment, the relevant comparison is between clinical-factor strata among patients receiving sutures, rather than simply between sutures and another treatment. Studies reporting only the frequency of a factor among all treated patients were not interpreted as demonstrating an association with failure. Mixed delivery-route cohorts were eligible for direct synthesis only when cesarean-specific factor-outcome information could be separated.

**Table 1:** Eligibility criteria based on the PICOS framework PICOS: Population, Intervention/Exposure, Comparator, Outcomes, and Study design; PPH: postpartum hemorrhage.

Domain	Inclusion criteria	Exclusion criteria
Population	Established PPH treated during cesarean delivery	Vaginal-only populations
	Extractable cesarean subgroups	Mixed routes without separable data
		Prophylaxis without established hemorrhage
Intervention or exposure	Therapeutic uterine compression sutures	Routine hysterotomy closure
	Maternal, obstetric, placental, physiological, or treatment-timing factors	Non-compression techniques
		Predictors of PPH occurrence alone
Comparator	Successful versus failed treatment	Single cases
	Clinical-factor strata with linked outcomes	Overall success rate without a factor–failure link
	Reconstructable patient-level comparisons	
Outcomes	Escalation after sutures	Only subsequent pregnancy, menstruation, or technical feasibility
	Immediate hemorrhage-related hysterectomy	
	Delayed hysterectomy and morbidity (assessed separately)	
Study design	Peer-reviewed cohorts or case-control studies	Reviews, meta-analyses, editorials, letters
	Trials with relevant prognostic analyses	Protocols, conference abstracts, preprints, theses
	Consecutive series with usable patient-level data	Isolated case reports
Other limits	No initial publication-date or language restriction	Unverifiable publication status
	Verifiable journal publication	Duplicate data for the same outcome

Information Sources and Search Strategy

PubMed/MEDLINE (Medical Literature Analysis and Retrieval System Online), Embase, Scopus, and Web of Science were searched without a lower publication-date restriction, combining postpartum hemorrhage terms with terms for uterine compression sutures and named techniques, such as B-Lynch [5], and those by Hayman et al. [6] and Cho et al. [7]. Failure and cesarean terms were deliberately not made mandatory, because relevant subgroup data often appeared only in full texts. Publication details and DOI-to-title correspondence were verified against bibliographic records and publisher pages through September 15, 2026. A planned five-year restriction, applicable only if more than 10 studies were eligible, was not applied. The search terms used for each database are given in the Appendices.

Selection Process

One author (FYA) screened titles and abstracts after removal of duplicate records, retrieved potentially relevant reports, and assessed their full texts against the PICOS criteria. Eligibility required therapeutic suturing for established hemorrhage and usable cesarean-specific clinical-factor and outcome information. Review articles, editorials, opinion letters, and other non-original publications were excluded from the primary evidence synthesis.

Data Collection and Data Items

Data extraction recorded study identifiers, design, setting, recruitment period, sample size, delivery route, suture technique, preceding and subsequent interventions, clinical-factor definitions, and outcomes. Identifiable vaginal-delivery cases were removed from cesarean subgroup calculations, reconstructed values were labeled separately from estimates reported by the original investigators, and missing numerical details were not imputed.

Outcome Definitions and Effect Measures

Escalation was defined as an additional invasive hemostatic intervention after compression sutures. Hemorrhage-related hysterectomy was analyzed separately from delayed hysterectomy for infection or ischemic complications. Procedures performed before suturing were not counted as subsequent suture failure. Reported counts were retained, and simple subgroup percentages were calculated only when both the numerator and denominator were identifiable. Event proportions were presented with Wilson score 95% confidence intervals (CIs). Where a clinical factor was cross-tabulated against a linked outcome, an unadjusted risk ratio (RR) with a 95% CI was calculated (0.5 continuity correction for zero cells), with two-sided Fisher exact p-values. No adjusted association was reconstructed from aggregate demographic summaries. Total blood loss, transfusion, and intensive care admission were not treated as baseline predictors unless measurement before the outcome was established.

Risk of Bias Assessment

One author (FYA) assessed each included study using the Quality In Prognosis Studies (QUIPS) tool [11]. Its six domains address study participation, study attrition, prognostic-factor measurement, outcome measurement, study confounding, and statistical analysis and reporting. Domain-level and overall judgments were categorized as low, moderate, or high risk of bias. The author-completed judgments were visualized using the Risk-of-bias VISualization (robvis) tool [12]. Risk-of-bias findings informed interpretation of the prognostic evidence; no summed numerical quality score was used.

Synthesis Methods and Certainty

Findings were grouped by clinical factor, delivery-route applicability, outcome definition, and whether associations were adjusted. Reporting followed the principles of Synthesis Without Meta-analysis (SWiM) [13]. Factor-specific estimates were not pooled because the exposures differed (placenta previa versus suspected placenta accreta spectrum), events were sparse, and adjusted estimates were unavailable. Pooling would have combined clinically distinct exposures; therefore, it was restricted to an exploratory random-effects meta-analysis of event proportions for outcomes defined consistently across studies: immediate hemorrhage-related hysterectomy and escalation after sutures. Logit-transformed proportions were pooled using the DerSimonian-Laird estimator, and heterogeneity was described using Cochran’s Q and I², recognizing their imprecision with few studies. Calculations were performed in Python (SciPy) (Python Software Foundation, Wilmington, Delaware, United States). As the number of included studies was small, funnel plots and statistical tests of reporting bias were not performed. Certainty was considered qualitatively, without a formal certainty-of-evidence grade.

Results

The database search identified 1,010 records (PubMed 459, Embase 238, Scopus 164, and Web of Science 149). After removal of 636 duplicates, 298 of 374 records were excluded at title and abstract screening. The remaining 76 reports were all retrieved and assessed. Of these, 72 were excluded (21 did not meet the inclusion criteria, 27 involved vaginal-only populations, and 24 were review articles, editorials, or opinion letters), leaving four included studies (Figure 1).

**Figure 1:**
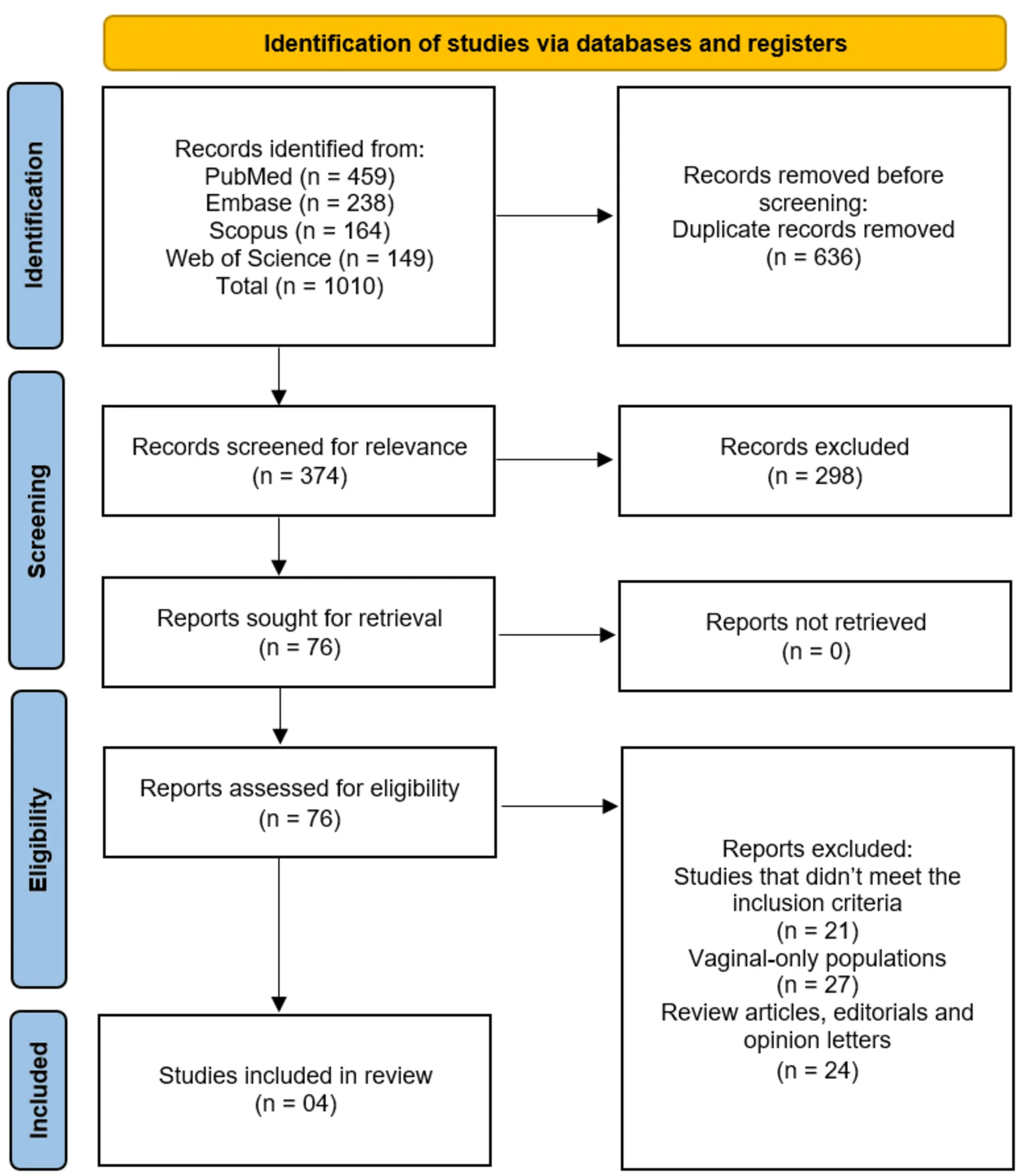
PRISMA flow diagram of study identification, screening, eligibility assessment, and inclusion PRISMA: Preferred Reporting Items for Systematic Reviews and Meta-Analyses.

Study Characteristics

The included studies were reported by Wohlmuth et al. [14], Sentilhes et al. [15], Chai and To [16], and Kojima et al. [17]. Together, their original cohorts contained 105 women; exclusion of three identifiable vaginal deliveries yielded 102 cesarean cases. Table 2 summarizes the study designs, populations, treatments, comparators, and principal findings.

**Table 2:** Characteristics of directly relevant primary studies PPH: postpartum hemorrhage.

Study	Country	Design and population	Sample	Intervention and comparator	Outcomes and findings
Wohlmuth et al. [14]	United States	Retrospective series; 1997–2005; refractory PPH at cesarean.	22	B-Lynch, alone or with vessel ligation; etiology subgroups.	5 hysterectomies; 85% success reported for atony. Exact factor-stratum denominators unavailable in abstract.
Sentilhes et al. [15]	France	Retrospective cohort; 2003–2007; persistent bleeding after devascularization.	15 total; 13 cesarean	B-Lynch after vessel ligation; reconstructed clinical-factor strata.	Cesarean subset: 2 immediate hysterectomies; 1 additional delayed hysterectomy.
Chai and To [16]	Hong Kong, China	Retrospective audit; 2008–2012; severe PPH.	35 total; 34 cesarean	B-Lynch/Bhal; etiology strata reconstructed after removing vaginal case.	Cesarean subset: 11 escalations; 8 hysterectomies.
Kojima et al. [17]	Japan	Retrospective cohort; January 2011–July 2025; PPH during cesarean.	33	Compression sutures; suspected accreta spectrum versus other cases.	10 escalations: 3 embolizations, 7 hysterectomies.

Treatment Outcomes

Table 3 separates escalation, immediate hysterectomy, and delayed uterine loss, with 95% CIs. Immediate hemorrhage-related hysterectomy ranged from 15.4% to 23.5% across the four studies, and escalation from 15.4% to 32.4% across the three studies reporting it. In the exploratory meta-analysis (Figure 2), the pooled proportion of immediate hemorrhage-related hysterectomy was 21.7% (95% CI 14.7-30.8%; 22/102; I² = 0%), and that of escalation was 29.3% (95% CI 20.3-40.3%; 23/80; I² = 0%). These pooled values describe the burden of failure among treated women; they do not identify which women failed.

**Table 3:** Outcome definitions and cesarean-specific events Pooled estimates: random-effects (DerSimonian-Laird) meta-analysis of logit-transformed proportions. CI: confidence interval.

Study	Cesarean, N	Escalation after sutures, n/N (%); 95%CI, Wilson score	Immediate hemorrhage-related hysterectomy, n/N (%); 95%CI, Wilson score	Delayed hysterectomy
Wohlmuth et al. [14]	22	Not separately reported	5/22 (22.7%); 95%CI, 10.1–43.4	Not established
Sentilhes et al. [15]	13	2/13 (15.4%); 95%CI, 4.3–42.2	2/13 (15.4%); 95%CI, 4.3–42.2	1/13 at six weeks; separate safety endpoint
Chai and To [16]	34	11/34 (32.4%); 95%CI, 19.1–49.2	8/34 (23.5%); 95%CI, 12.4–40.0	Not separately quantified here
Kojima et al. [17]	33	10/33 (30.3%); 95%CI, 17.4–47.3	7/33 (21.2%; 95%CI, 10.7–37.8)	Not established
Exploratory pooled estimate	80–102	29.3% (95%CI, 20.3–40.3); I² = 0%	21.7% (95%CI, 14.7–30.8); I² = 0%	Not pooled

**Figure 2:**
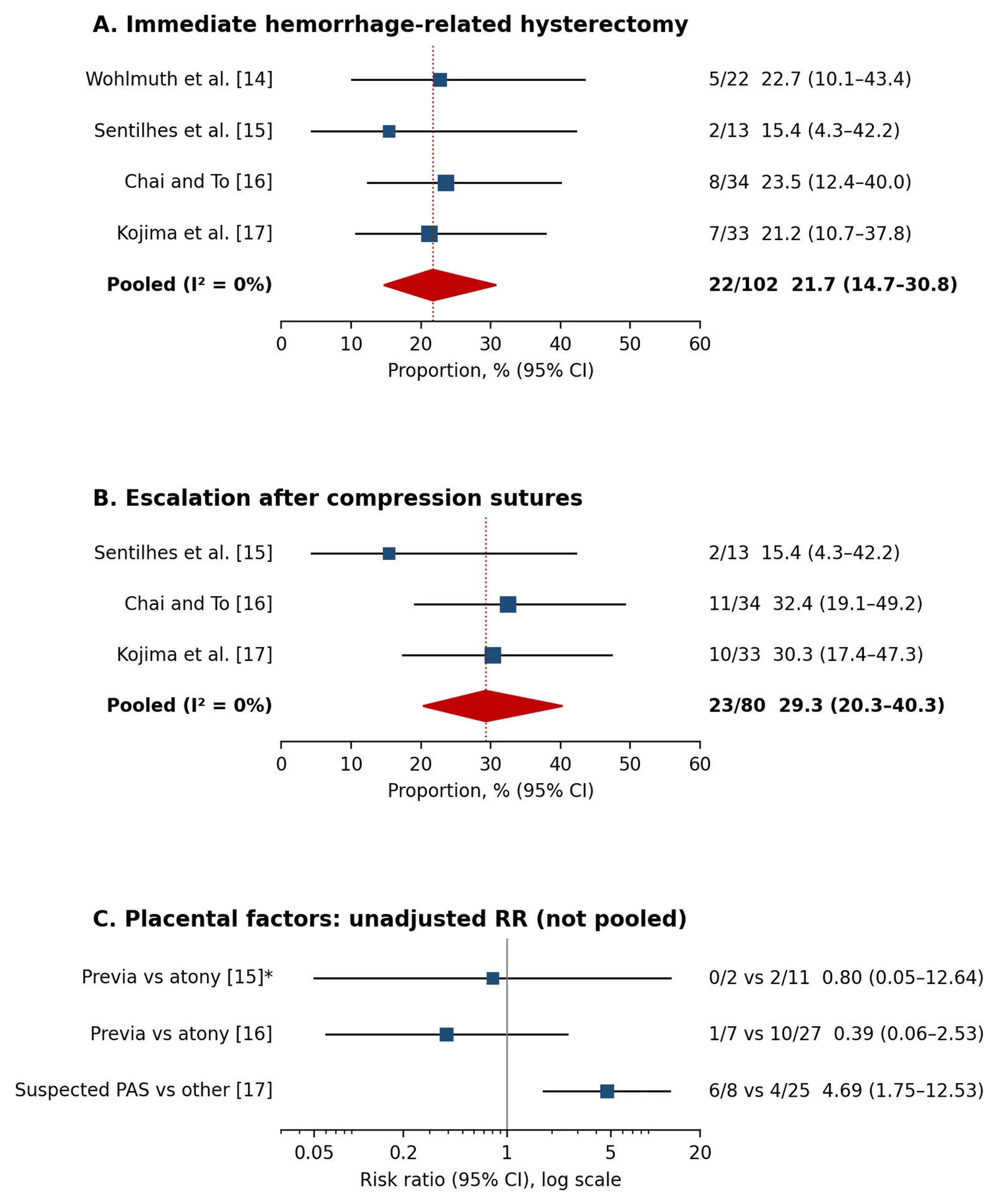
Study-level and exploratory pooled estimates of compression-suture failure during cesarean delivery (A) Immediate hemorrhage-related hysterectomy; (B) escalation after compression sutures; (C) unadjusted risk ratios for placental factors, not pooled. Squares are study estimates with Wilson 95% CIs; diamonds are random-effects (DerSimonian-Laird) pooled proportions. *0.5 continuity correction. CI: confidence interval; RR: risk ratio; PAS: placenta accreta spectrum.

Clinical Factors

Table 4 presents the available factor contrasts with unadjusted RRs, 95% CIs, and the origin of each estimate (reported by the original investigators or reconstructed by the review). Suspected placenta accreta spectrum was the only factor with a clearly distinguishable contrast (escalation RR 4.69, 95% CI 1.75-12.53; p = 0.004) [17], whereas placenta previa versus atony yielded imprecise estimates in both studies that permitted the comparison [15,16] (Figure 2C). These small, unadjusted strata cannot establish independent predictors, and no pooled factor-specific effect was calculated.

**Table 4:** Clinical-factor evidence within the directly relevant reports RR expresses the risk in the first-named group relative to the second. *0.5 continuity correction applied for a zero cell. p-values: two-sided Fisher exact test. RR: risk ratio; CI: confidence interval.

Factor and source	Comparison (events/N)	Unadjusted RR (95% CI); p	Data source	Interpretation
Hemorrhage etiology [14]	Atony success 85%; overall success 77%.	Not calculable	Reported (abstract)	No exact non-atony denominator or adjusted effect recoverable from abstract.
Previa versus atony [15]	Immediate hysterectomy: 0/2 versus 2/11.	0.80 (0.05–12.64)*; p = 1.00	Reconstructed from individual cesarean records	Extremely sparse.
Previa versus atony [16]	Escalation: 1/7 versus 10/27.	0.39 (0.06–2.53); p = 0.38	Reconstructed after removing vaginal case	Previa is not equivalent to accreta spectrum.
Suspected accreta spectrum versus other [17]	Escalation: 6/8 (75.0%) versus 4/25 (16.0%).	4.69 (1.75–12.53); p = 0.004	Counts and p reported; RR calculated by review	Unadjusted descriptive contrast.
Age, parity, timing, and coagulation status	No robust cesarean-specific adjusted association verified.	Not estimable	—	Absence of adequate evidence does not demonstrate absence of an effect.

The findings do not show a uniform disadvantage for every form of placental bleeding. Differences in placental diagnosis, suture placement, and treatment selection prevent a common interpretation across the etiology strata. Higher overall morbidity after unsuccessful treatment is also not proof that those same measurements could have predicted failure before suturing (Figure 2).

Risk of Bias

Figure 3 presents the author-completed QUIPS assessments. Sentilhes et al. [15] and Chai and To [16] were rated at low risk in all six domains and at low overall risk. Wohlmuth et al. [14] and Kojima et al. [17] were rated at moderate risk for study participation and prognostic-factor measurement, low risk for attrition and outcome measurement, and high risk for confounding and statistical analysis and reporting, resulting in high overall risk. Thus, two of the four studies had low overall risk, and two had high overall risk. These judgments describe study-level bias and do not remove the limitations imposed by sparse events or establish independent prediction.

**Figure 3:**
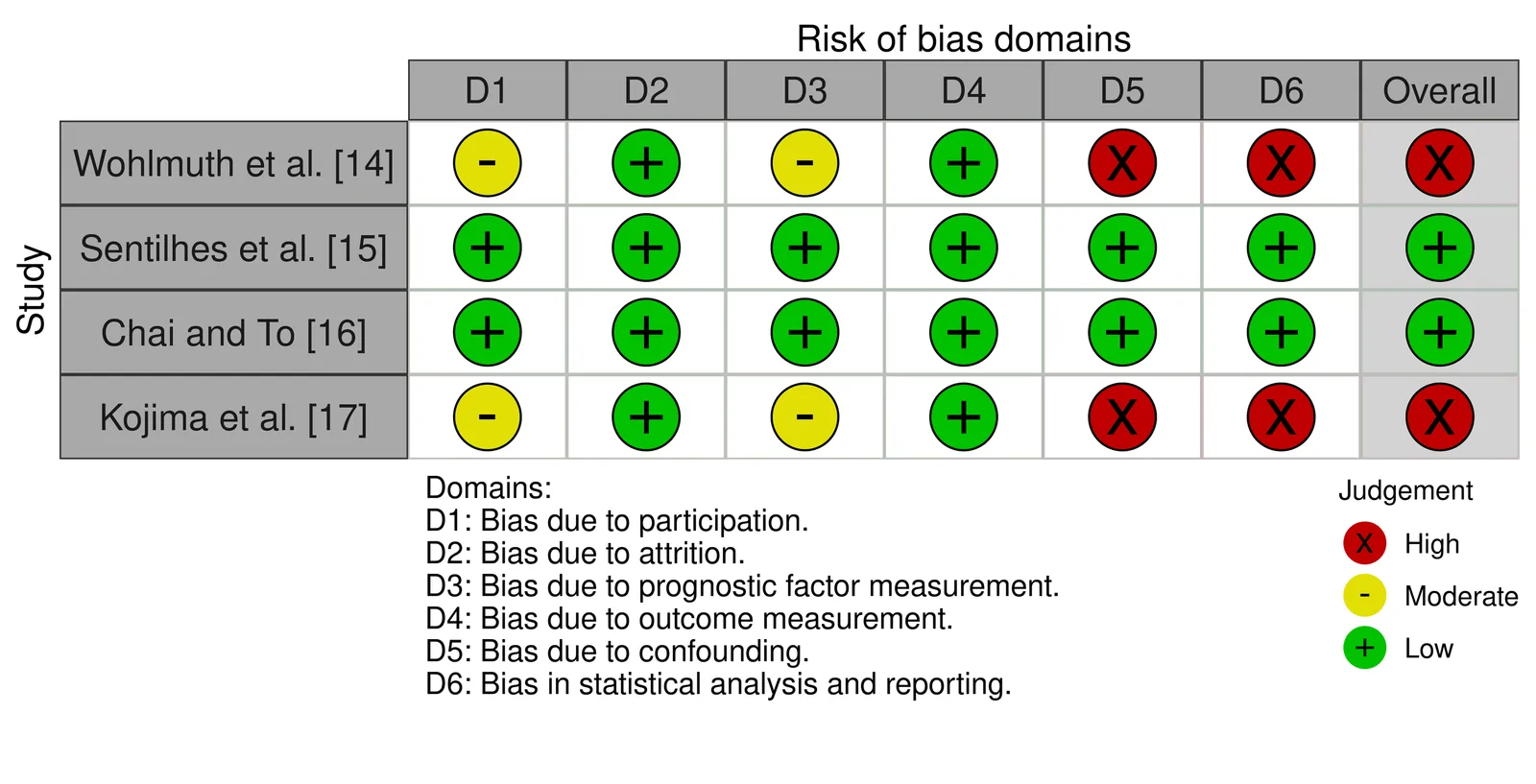
QUIPS risk-of-bias judgments Green (+), low risk; yellow (−), moderate risk; red (×), high risk QUIPS: Quality In Prognosis Studies; robvis: Risk-of-bias VISualization.

Discussion

Principal Findings

The central finding is the limited amount of directly interpretable evidence on which clinical factors predict failure of uterine compression sutures during cesarean delivery. Many published studies report successful compression-suture procedures, but they provide little information on which women are likely to experience failure. To identify such women, studies must compare clinical factors between patients in whom the sutures succeeded and those in whom they failed. Reporting that many treated women were multiparous, anemic, or delivered by emergency cesarean does not provide such a comparison. Without an appropriate denominator in the successful and failed groups, these observations describe the treated population rather than explain variation in outcome. This distinction should guide both interpretation of the present evidence and the design of future studies. The exploratory pooled estimates indicate that about one in five treated women underwent immediate hysterectomy, but they quantify the burden of failure rather than its determinants. Here, direct evidence from the four included cesarean-specific studies [14-17] is distinguished from contextual evidence [18-28], which derives from mixed delivery-route cohorts, other hemostatic interventions, or non-prognostic designs and is used to generate hypotheses rather than to estimate effects.

Placental Pathology

The strongest directly relevant signal concerns placental pathology, although the current findings do not support treating placenta previa and placenta accreta spectrum as interchangeable. Suspected invasive adherence may indicate a different source and extent of bleeding from a low placental implantation without invasion. The available direct comparisons are too small and heterogeneous to establish a universal effect size [14-17]. Clinically, the useful implication is to precisely characterize the bleeding source. A diagnostic category should inform evaluation of the anatomy and available treatment options; however, such data cannot justify a general rule that every patient in a particular category should bypass a uterus-preserving intervention.

Management of placenta accreta spectrum also involves treatment strategies that cannot be reduced to a comparison of successful and unsuccessful sutures. In the PACCRETA prospective study, conservative management with the placenta left in situ and cesarean hysterectomy were associated with different profiles of transfusion and later morbidity [23]. That comparison addresses a planned management strategy, whereas emergency suturing after placental separation addresses active bleeding in a different selected population. The relevance to this review is the importance of documenting whether invasive placentation was suspected antenatally, recognized intraoperatively, or diagnosed after hysterectomy. These groups may differ in preparation, operative intent, and verification of the diagnosis.

Timing of Intervention

The larger United Kingdom Obstetric Surveillance System study by Kayem et al. provides important contextual evidence [18]. Among 211 women receiving compression sutures, failure leading to hysterectomy occurred in approximately one quarter; a delay of two to six hours was independently associated with increased odds of hysterectomy. Its mixed delivery-route population is crucial to interpretation. Access to the uterus after vaginal birth, the need to arrange laparotomy, and the course of hemorrhage before surgery differed from the circumstances of an open cesarean procedure. The study supports concern about delayed control of bleeding, but its overall association cannot be relabeled a validated cesarean-only timing threshold.

Coagulation Factors

Coagulation deserves particular attention because it may influence the ability of mechanical pressure to produce durable hemostasis. Charbit and colleagues linked early fibrinogen depletion with progression to severe PPH [21]. Collins and colleagues prospectively examined fibrin-based clot formation and fibrinogen as biomarkers of hemorrhage progression [22]. These studies support measuring the physiological state of the patient during an evolving bleed; they do not establish a fibrinogen cutoff that predicts suture failure. A future suture cohort would need to record the time of the sample relative to placement, transfusion, and escalation. Otherwise, a low value obtained after prolonged uncontrolled bleeding could be mistaken for a pre-existing predictor.

The same temporal problem applies to total blood loss and transfusion volume. Both can increase because sutures fail. If data are collected at the end of surgery and placed in a model intended to guide the decision before suturing, the model will incorporate information that was unavailable at the decision point. Its apparent predictive performance would therefore be misleading. Studies should distinguish blood loss accumulated before placement from subsequent loss and should record the rate of ongoing bleeding, not just the final total. Transfusion should likewise be separated into products given before and after the attempted procedure. These requirements follow from the prognostic question rather than from any claim that a particular numerical threshold is already established.

Contextual Evidence From Other Interventions and Techniques

Kong and To compared balloon tamponade with compression sutures and examined prognostic factors across the combined treatment population [19]. Greater blood loss before intervention, accreta, and coagulopathy were associated with poorer outcomes, but the regression was not confined to cesarean patients receiving sutures. This illustrates a common applicability problem: a model may be valid for a broader clinical pathway while answering a different question from the one posed here. Liu and colleagues similarly investigated predictors of balloon tamponade failure [20]. Such studies can suggest factors worth measuring, but neither the direction nor the magnitude of a balloon-specific association should be transferred directly to compression sutures.

Technique development offers plausible ways to improve the anatomical match between a suture and the bleeding source. Han et al. reported a step-wise knapsack-like technique [26], while Li et al. described mesh compression suturing [27]. These reports contribute information about feasibility and outcomes of newer approaches, but do not provide a validated clinical-factor model for failure. The distinction matters when an innovative series reports very few failures: a high observed success rate may be encouraging, yet it supplies little statistical information about differential risk. Comparing it with an older rescue cohort without accounting for case severity, exclusions, and treatment sequence would not establish superiority.

The prospective series by Devi et al. further illustrates why a technical success report must be read carefully before extracting predictors [28]. Its aggregate descriptions and qualitative observations cannot supply cesarean-specific adjusted associations. A factor may be mentioned in a discussion because it was reported elsewhere, rather than because the investigators measured and tested it in their own cohort. For evidence synthesis, each association must therefore be traced to its own study population, denominator, outcome, and analysis. Citations that merely repeat an earlier observation should not be counted as independent confirmation. This is especially relevant in a small literature where the same older reports are cited repeatedly.

Outcome Definitions

The definition of failure determines what a prognostic factor means. A composite of embolization, vessel ligation, and hysterectomy captures escalation, but each component has a different consequence for the patient and is influenced by local availability. A center with round-the-clock interventional radiology may preserve the uterus after unsuccessful suturing in situations where another center proceeds directly to hysterectomy. Consequently, a lower hysterectomy rate does not necessarily indicate a more effective initial suture. Reporting both immediate control without additional treatment and final uterine preservation is necessary to separate procedural performance from the effectiveness of the overall rescue pathway.

Delayed uterine loss should also remain distinct from immediate failure to arrest bleeding. A procedure can achieve initial hemostasis yet be followed by infection, ischemic injury, or another complication requiring later surgery. Reproductive follow-up research, including Fuglsang’s study [24], addresses an additional dimension of outcome beyond survival of the index operation. Kwong and colleagues examined menstrual, fertility, and psychological outcomes after compression sutures [25]. Such follow-up is valuable but is vulnerable to selection when only women whose uterus was preserved enter the reproductive analysis. Future studies should retain the full treated cohort for acute outcomes and then state explicitly which survivors were eligible and available for longer-term assessment.

Future Research Needs

Selection into treatment is another major concern. Women with the most extensive placental invasion or profound instability may undergo hysterectomy without an attempted suture. Even within cases using sutures, some centers may use sutures early for readily compressible atony, whereas others reserve them for the last stage after several interventions. An analysis restricted to treated women is appropriate for the stated prognostic question, but its findings apply to that selected group. A low failure rate cannot be generalized to patients who were never considered suitable for suturing. Transparent reporting of all severe PPH cases, reasons for choosing sutures, and reasons for proceeding directly to definitive surgery would improve interpretation.

An adequately designed prognostic study should define the moment at which prediction is intended. One useful time point is immediately before placement of the first compression suture; another is reassessment shortly after placement. These are different models with different eligible variables. The first might evaluate diagnosis, existing physiological disturbance, prior treatment, and elapsed time. The second could incorporate response to compression and persistent bleeding after placement. Combining the two time points without distinction obscures the intended clinical use. A risk estimate is useful only when it can be calculated from information available when the decision must be made.

Future multicenter cohorts should use a common minimum dataset and retain continuous measurements wherever feasible. Arbitrary age, blood loss, and laboratory cutoffs can discard information and produce unstable results in small samples. The number of failures, rather than merely the total sample size, constrains the complexity of analysis. Investigators should avoid extensive stepwise selection in sparse datasets, describe missing data, and account for hospital-level practice. Any proposed model should report calibration as well as discrimination and undergo validation in a separate population. A statistically significant factor in one small cohort is insufficient to establish either reliable individual prediction or an improvement in clinical decisions.

A study that does not identify a significant association may be underpowered; absence of statistical significance therefore does not establish absence of a clinically relevant relationship. Similarly, overall risk-of-bias judgments and the amount of usable prognostic information address different questions. A study with reliable outcome recording may still provide only a small number of events or no precise adjusted association. These distinctions are essential when interpreting the present evidence. Placental diagnosis, treatment timing, and physiological deterioration merit investigation in larger cohorts, while their independent predictive contribution specifically after cesarean compression suturing remains insufficiently established.

Implications for Practice

For practice, these findings favor structured documentation and timely reassessment within an established hemorrhage response system. They do not justify prolonging an unsuccessful attempt to preserve the uterus, nor do they support a new mandatory treatment threshold. The immediate objectives remain control of bleeding and maternal stabilization, with uterine preservation pursued when compatible with those objectives. Research should evaluate whether a proposed prognostic assessment improves escalation decisions and maternal outcomes rather than merely whether it predicts a composite endpoint. That patient-centered standard would make the next generation of studies more informative than another uncontrolled percentage of successful procedures.

Limitations

The evidence is limited by four small observational studies, sparse failure events, different rescue pathways, historical changes in practice, and inconsistent timing of prognostic-factor measurements. Two studies were rated at high overall risk of bias. Low-risk ratings in the other studies do not establish precision, generalizability, or a validated prediction model. Screening and risk-of-bias assessment were performed by a single review author, making selection and judgment errors possible. Reconstructed cesarean subgroups are descriptive secondary extractions rather than prospectively specified analyses by the original investigators. Abstract-level numerical summaries supplied some extracted findings, limiting the detail available for those comparisons. The pooled proportions were exploratory and based on three or four studies, so heterogeneity statistics were imprecise, and these estimates should not be read as benchmark failure rates. No pooled factor-specific effect estimate or formal certainty grade was produced.

## Conclusions

Evidence for clinical factors associated with compression suture failure during cesarean delivery is limited. In treated cesarean cohorts, about one in five women required immediate hemorrhage-related hysterectomy. Placental pathology is a directly observed source of variation, but suspected placenta accreta spectrum should not be conflated with placenta previa, and no robust cesarean-specific prediction rule has been established. Additional hemostatic treatment, immediate hysterectomy, and delayed uterine loss represent distinct outcomes. The findings support careful characterization of hemorrhage etiology and timely reassessment within established obstetric hemorrhage pathways.

## Appendices

**Table 5:** Search expressions by database MeSH: Medical Subject Headings; tiab: title/abstract; exp: exploded subject heading; ti: title; ab: abstract; TITLE-ABS-KEY: title, abstract, and keywords; TS: Topic (In Web of Science, Topic also includes Keywords Plus.)

Database and interface	Search expression
PubMed/MEDLINE Author-supplied expression	("Postpartum Hemorrhage"[Mesh] OR postpartum hemorrhag*[tiab] OR postpartum haemorrhag*[tiab] OR post-partum hemorrhag*[tiab] OR post-partum haemorrhag*[tiab]) AND ("uterine compression"[tiab] OR "compression suture"[tiab] OR "compression sutures"[tiab] OR "B-Lynch"[tiab] OR "B Lynch"[tiab] OR Hayman[tiab] OR "brace suture"[tiab] OR "brace sutures"[tiab] OR "Cho suture"[tiab] OR "Cho sutures"[tiab] OR "Pereira suture"[tiab] OR "Pereira sutures"[tiab] OR "Matsubara-Yano"[tiab] OR "uterine sandwich"[tiab] OR "hemostatic suturing"[tiab] OR "haemostatic suturing"[tiab])
Embase.com Equivalent formulation	('postpartum hemorrhage'/exp OR ('postpartum hemorrhag*' OR 'postpartum haemorrhag*' OR 'post-partum hemorrhag*' OR 'post-partum haemorrhag*'):ti,ab) AND ('uterine compression' OR 'compression suture' OR 'compression sutures' OR 'B-Lynch' OR 'B Lynch' OR 'Hayman' OR 'brace suture' OR 'brace sutures' OR 'Cho suture' OR 'Cho sutures' OR 'Pereira suture' OR 'Pereira sutures' OR 'Matsubara-Yano' OR 'uterine sandwich' OR 'hemostatic suturing' OR 'haemostatic suturing'):ti,ab
Scopus Equivalent formulation	TITLE-ABS-KEY("postpartum hemorrhag*" OR "postpartum haemorrhag*" OR "post-partum hemorrhag*" OR "post-partum haemorrhag*") AND TITLE-ABS-KEY("uterine compression" OR "compression suture" OR "compression sutures" OR "B-Lynch" OR "B Lynch" OR "Hayman" OR "brace suture" OR "brace sutures" OR "Cho suture" OR "Cho sutures" OR "Pereira suture" OR "Pereira sutures" OR "Matsubara-Yano" OR "uterine sandwich" OR "hemostatic suturing" OR "haemostatic suturing")
Web of Science Core Collection Equivalent formulation	TS=("postpartum hemorrhag*" OR "postpartum haemorrhag*" OR "post-partum hemorrhag*" OR "post-partum haemorrhag*") AND TS=("uterine compression" OR "compression suture" OR "compression sutures" OR "B-Lynch" OR "B Lynch" OR "Hayman" OR "brace suture" OR "brace sutures" OR "Cho suture" OR "Cho sutures" OR "Pereira suture" OR "Pereira sutures" OR "Matsubara-Yano" OR "uterine sandwich" OR "hemostatic suturing" OR "haemostatic suturing")
